# Impact on Antibiotic Resistance, Therapeutic Success, and Control of Side Effects in Therapeutic Drug Monitoring (TDM) of Daptomycin: A Scoping Review

**DOI:** 10.3390/antibiotics10030263

**Published:** 2021-03-05

**Authors:** Carolina Osorio, Laura Garzón, Diego Jaimes, Edwin Silva, Rosa-Helena Bustos

**Affiliations:** 1 Evidence-Based Therapeutics Group, Clinical Pharmacology, Universidad de La Sabana, Chía 140013, Colombia; carolinaosre@unisabana.edu.co (C.O.); lauragapa@unisabana.edu.co (L.G.); diegojf@unisabana.edu.co (D.J.); 2 Faculty of Medicine, University of La Sabana, Chía 140013, Colombia; edwin.silva@unisabana.edu.co

**Keywords:** therapeutic drug monitoring (TDM), daptomycin, Gram-positive bacteria, antimicrobial resistance, patient in critical condition

## Abstract

Antimicrobial resistance (AR) is a problem that threatens the search for adequate safe and effective antibiotic therapy against multi-resistant bacteria like methicillin-resistant *Staphylococcus aureus* (MRSA), and vancomycin-resistant *Enterococci* (VRE) and *Clostridium difficile*, among others. Daptomycin is the treatment of choice for some infections caused by Gram-positive bacteria, indicated most of the time in patients with special clinical conditions where its high pharmacokinetic variability (PK) does not allow adequate plasma concentrations to be reached. The objective of this review is to describe the data available about the type of therapeutic drug monitoring (TDM) method used and described so far in hospitalized patients with daptomycin and to describe its impact on therapeutic success, suppression of bacterial resistance, and control of side effects. The need to create worldwide strategies for the appropriate use of antibiotics is clear, and one of these is the performance of therapeutic drug monitoring (TDM). TDM helps to achieve a dose adjustment and obtain a favorable clinical outcome for patients by measuring plasma concentrations of an administered drug, making a rational interpretation guided by a predefined concentration range, and, thus, adjusting dosages individually.

## 1. Introduction

The World Health Organization (WHO) has reviewed in detail the increase in antimicrobial resistance (AR), which represents a threat to health worldwide [1]. According to the report on AR, this problem is among the top 10 public health threats and is related to the inappropriate use of antibiotics [2]. Due to the great spread of multi-resistant (MDR) and pan-resistant bacteria that have acquired various multi-resistance mechanisms, these are compromising the ability of antibiotics to control infection and produce a favorable clinical outcome for patients [3].

Due to this situation, the WHO has classified MDR bacteria into two different groups according to the priority regarding antimicrobial therapy and the development of new antibiotics [4]. A list of antibiotic resistant bacteria was reported, describing some Gram-positive bacteria such as Methicillin-resistant *Staphylococcus aureus* (MRSA) and vancomycin-resistant *Enterococci* (VRE) and *Clostridium difficile.* These microorganisms comprise 7 of the 18 urgent and serious AR-related threats described by the United States Centers for Disease Control and Prevention (CDC) [5], and they are one of the main risk factors associated with in-hospital mortality [6,7,8]. For this reason, it has been necessary to develop strategies that allow an early diagnosis, focusing on the identification of the causative microorganism and initiating a timely and adequate anti-infective treatment to reduce the risk of mortality and increase rates of therapeutic success [7,8]. Infections caused by Gram-positive bacteria are resistant to multiple antibiotics [9], and despite their frequency, there are few studies that report the best management practices for these infections, for which the WHO has created campaigns focused on the research and development of new antibiotics [1]. Currently, there are two antibiotics approved by the US Food and Drug Administration (FDA) for the treatment of MRSA bacteremia and endocarditis: vancomycin and daptomycin [10].

Daptomycin is categorized as one of the latest antibiotics developed for the management of serious infections caused by multi-resistant germs, especially Gram-positive bacteria. Additionally, it has been shown that despite low doses, it could develop severe side effects, which is why performing therapeutic drug monitoring (TDM) would be ideal to control complications and maintain doses within therapeutic ranges.

### 1.1. Structure and Mechanism of Action of Daptomycin

Daptomycin is a cyclic lipopeptide antibiotic that is structurally and functionally related to cationic antimicrobial peptides produced by the innate immune system. The daptomycin molecule consists of a cyclic polypeptide nucleus (tridecapeptide) of 13 amino acids, six of which are non-proteinogenic: D-Asn, ornithine (Orn), D-Ala, D-Ser, (2S, 3R)-methylglutamate (MeGlu), and kynurenine (Kyn) [11]. The 10 terminal carbon residues form a closed macrocyclic ring via an ester bond and an exocyclic side chain of 3 amino acids with a terminal tryptophan (Thr) linked to a decanoic acid residue [12], and it contains an altered sequence of the N-terminal decanoyl fatty acid lethal chain [13].

Regarding its mechanism of action, daptomycin is a calcium-dependent antibiotic (CDA), which results in membrane depolarization and loss of intracellular components, such as K^+^, Mg^2+^, and ATP [14], which is attractive by not causing bacterial lysis [15]. The groups responsible for this activity are the core macrocycle (binding of an ester bridge) and the group containing tsushimycin (amphomycin, laspartomycin, and several others). Likewise, these groups share the mechanism of action, which consists of binding and sequestering a constituent of the bacterial membrane (undecaprenol phosphate), which is important in the transport of coenzymes and in the assembly and translocation of peptidoglycan precursors in the plasma membrane [11].

### 1.2. Pharmacokinetic and Pharmacodynamic Considerations

Daptomycin exhibits the following pharmacokinetic/pharmacodynamic (PK/PD) characteristics: (1) hydrophilic drug [16,17]; (2) high binding to plasma proteins (92–94%); (3) small volume of distribution (Vd) (0.1 L/kg); (4) renal elimination [6,16]; (5) elimination half-life (V_1/2_) of 8 to 9 h; and (6) post-antibiotic effect up to 6.8 h [17,18]. This antibiotic is used mainly for the management of bacteremia, complicated skin and soft tissue infections, and endocarditis secondary to Gram-positive bacteria [8,15,18]. The PK/PD index allows us to analyze the relationship between dose and effect, taking into account plasma concentration as an important variable when relating drug exposure with the minimum inhibitory concentration (MIC) of the pathogen. Based on this, antimicrobials are classified by the following PK/PD indices that describe their efficacy: (1) time-dependent—the drug concentration remains above the MIC in a dose interval (fT > MIC); (2) concentration-dependent—the maximum concentration peak remains above the MIC (C_max_/MIC); and (3) area under the curve—the concentration and time remain above the MIC for at least 24 h (AUC_0–24_/MIC) [6,7,19]. The bactericidal effect of daptomycin is associated with an AUC/MIC > or equal to 200 [7], but recent studies have shown greater therapeutic efficacy with AUC/MIC levels > 666 [6,7,20] and a C_max_/MIC between 12–94 to obtain an optimal bacteriostatic effect [7] and suppression of bacterial resistance [19]. Therapeutic efficacy is directly related to optimal serum concentrations [19,20], which are expected to be obtained by means of a standard dosage schedule.

When we face patients with special clinical conditions (sepsis, obesity, chronic kidney disease, renal replacement therapy (RRT), and hypoalbuminemia), it is not so feasible to obtain adequate plasma levels because they present pathophysiological changes altering the PK of medications, leading to a variable exposure and obtaining sub or supraoptimal concentrations despite receiving the recommended doses [7,19,21,22]. Therefore, it is essential to optimize the use of antibiotics in these types of patients, performing individual dosage regimens to maximize efficacy, minimize toxicity, and achieve PK/PD goals [7,8,16,19]. A method that can contribute to the rational individualization of pharmacological treatments and the evolution of personalized medicine [23,24] is therapeutic drug monitoring (TDM), which involves the following: (1) the measurement of plasma concentrations of some drugs or in different biological fluids (plasma, serum, urine, saliva, etc.) of patients [7]; (2) dose individualization based on circulating plasma exposure, targeting a predefined concentration range [24]; and (3) maintaining blood levels within the therapeutic window to achieve PK/PD objectives and thus increase the probability of achieving therapeutic success and reduce the risk of toxicity and development of bacterial resistance [8,19,25,26].

TDM is a method that allows the plasma concentrations of an administered drug to be measured, and then a rational interpretation can be made, guided by a predefined concentration range, and thus the doses can be adjusted individually [21,24]. Initially, TDM was used only to reduce the risk of toxicity of some specific medications, especially those with a narrow therapeutic index (NTI) [6,26,27]; however, taking into account the possible changes that a drug may undergo in plasma concentration after administration and its close relationship with pharmacological effects, it is considered that it can also help to achieve PK/PD goals and thus improve the therapeutic efficacy of many other drugs, such as antibiotics [6,25,26].

TDM allows to one differentiate the clinical symptoms and signs secondary to toxicity due to overdose, compared with the clinical condition that can be easily confused [24]. However, the rational interpretation of TDM and the consequent dose adjustment require knowledge of the following: (1) availability of a precise and selective bioanalytical assay with a fast response time; (2) defined target concentration range to obtain drug response; (3) understanding of the PK characteristics of the measured drug; (4) susceptibility of the causative bacterial pathogen [7,19,25]; (5) PK/PD variabilities of each patient according to their clinical condition; and (6) suspected drug interactions and side effects [23].

Despite the fact that TDM was initially used in drugs with NTI in order to reduce the risk of toxicity, it is considered that there is the possibility of a new use and approach given to the increase of multidrug-resistant germs associated with serious infections, which increasingly present limitation in therapeutic options. The objective of this review is to describe the data available about the type of TDM method used and described in hospitalized patients with daptomycin and to describe its impact on therapeutic success, suppression of bacterial resistance, and control of side effects.

## 2. Results

**Figure 1:**
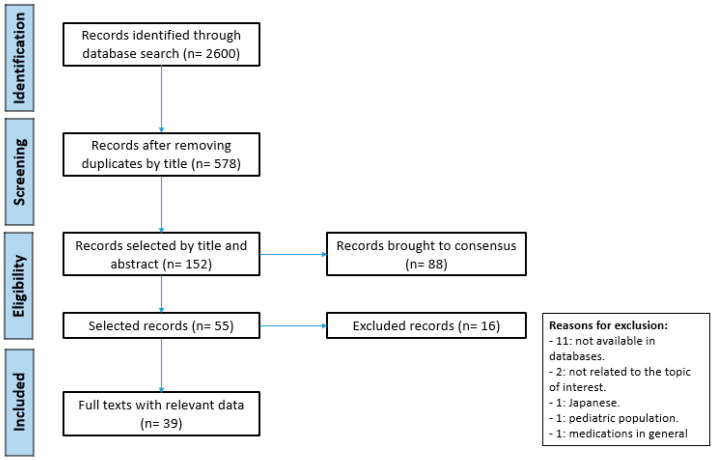
Study selection process according to PRISMA guidelines.

Initial database searches yielded 578 unique articles, of which after refining the search according to the exclusion criteria, 55 studies were reviewed in their entirety, 16 were excluded, and 39 met the inclusion criteria (Figure 1).

Among the types of study design, the following were found: letter to an editor [22], poster [25,26], case report [27,28,29,30], bioanalytical methodology [8,16,20,31,32,33,34,35,36,37,38,39,40,41,42,43,44,45], review article [6,7,15,21,23,24,46,47,48], observational prospective study [17,18], observational retrospective study [49,50], and a single non-randomized clinical study [19]. The publication dates of the selected studies included publications from 2008 to 2020, and the type of design of each study obtained, date of publication, and general characteristics related to therapeutic monitoring are shown in Table 1.

### 2.1. Therapeutic Monitoring Methods

Publication dates ranged from 2008 to 2020, and various types of methods for TDM in hospitalized daptomycin patients were described. Among the most common bioanalytical methods we found was the use of liquid chromatography–tandem mass spectrometry (LC-MS/MS) [16,22,25,29,33,37,42,46], followed by high performance liquid chromatography (HPLC) [8,18,23,28,30,41,47], ultrahigh pressure liquid chromatography–tandem mass spectrometry method (UHPLC-MS/MS) [38,39,43,44], high performance liquid chromatography with ultraviolet detection (HPLC-UV) [17,38], dual use of high performance liquid chromatography with ultraviolet detection (HPLC-UV), and liquid chromatography–tandem mass spectrometry (LC-MS/MS) [6,7]. Other methods were also found, such as ultrahigh-performance liquid chromatography equipped with a photodiode array (UHPLC-PDA) [32,49], Bayesian estimation [21], and liquid chromatography using a core-layer octadecylsilyl microparticle coupled to tandem mass spectrometry [20]. Regarding clinical methods, dosing protocols were described [50], as were Monte Carlo simulations [43], and population PK models [46]. Additionally, there were five studies that described the importance of performing TDM in hospitalized patients with daptomycin to achieve therapeutic success, control of side effects, and control of bacterial resistance [19,27,32,34,49]. Table 2 summarizes the types of TDM methods found for daptomycin.

It was found that the average C_min_ and C_max_ described by the TDM methods was ~13.16 mg/L (7, 41, 45, 46, 49) and ~66.6 mg/L [8,16,42,46,50], respectively. The AUC/MIC results were variable depending on the administered dose. For doses of 6 mg/kg/day and 8 mg/kg/day, there was evidence of an average value of AUC/MIC of ~642.69 and ~788.85, respectively, levels higher than those recommended to obtain therapeutic efficacy. This shows us that patients with severe infections who received doses of 8 mg/kg/day have higher values of C_max_ and AUC/MIC compared to those who received doses of 6 mg/kg/day, having a greater probability of therapeutic success [19].

It should be noted that the blood samples studied in the different articles were taken from populations that were in different clinical conditions, but these results showed us in detail that the different bioanalytical methods used to perform TDM in patients hospitalized with daptomycin helped to optimize treatment and achieve therapeutic success in patients with Gram-positive bacterial infections.

### 2.2. Bacterial Resistance

Very little is known about the frequency of occurrence of Gram-positive bacteria resistant to daptomycin. However, over the years, some reports have appeared related to microorganisms such as *Staphylococcus aureus, Enterococcus faecium*, and *Enterococcus faecalis.* This resistance occurs mainly in the context of prolonged treatment and infections with a high bacterial load, but it can occur in the presence of previous exposure to daptomycin. It seems that resistance both in *Staphylococcus* spp. and *Enterococcus* spp. is mediated by adaptations to cell wall homeostasis and membrane phospholipid metabolism [3,51].

During the analysis of the articles, none of them showed development of bacterial resistance to daptomycin, but yet there was a need to increase the dose in those patients with bacteremia and persistent infections by Gram-positive bacteria. A case report describes a patient with bacteremia that persisted with positive blood cultures 14 days after starting treatment with daptomycin, which is why he required dose adjustment to up 14 mg/kg/day to achieve PK/PD goals and therapeutic efficacy [27].

### 2.3. Therapeutic Success and Control of Side Effects

It was possible to show that most of the methods described reached levels of AUC/MIC and C_max_/MIC, reaching daptomycin PK/PD goals [8,19,40,44,45,46,49] with doses of 6–8 mg/kg/day [17,23,42]. Additionally, it was found that for those bacteremia or persistent infections, the dose needed to be increased >8 mg/kg/day, related to an increase in the levels of C_min_ [18], but without development of side effects [17,18,22,46,51]. The C_min_ > 24.3 mg/L, has been associated with an elevation of creatine kinase (CPK) of up to 30 times [6,7,27], being the main marker of toxicity at the muscle level due to the use of daptomycin [35]. Patients with special clinical conditions, mentioned previously, require dose adjustment of daptomycin, since an increase in half-life could be evidenced (V_1/2_) as well as a decreased in renal clearance (CL), leading to an increase in plasma concentrations, and therefore, a high risk of developing side effects associated with elevated CPK [21,23,32]. One study showed an increase in CPK in approximately 43–64% of the study population, without subsequent complications [50]; therefore, daptomycin is an antibiotic candidate for TDM, especially for those patients with special clinical conditions due to its intra- and inter-individual variability of their PK/PD [18,48].

## 3. Discussion

Most of the articles selected within this review describe a significant benefit in terms of performing TDM for hospitalized patients with daptomycin, especially those with special clinical conditions (obesity, chronic kidney disease, hypoalbuminemia, renal replacement therapy). TDM studies have been carried out in this type of population because they present alterations in their PK, ignoring the behavior of the drug, and as a consequence presenting a change in plasma concentrations with a high risk of presenting therapeutic failure or toxicity [6,17,18,22,35,36,52]. The interest in this group of patients is based on the great burden of infection presented by Gram-positive bacteria as the causative agent, being closely related to an increase in mortality [6,15]. Hematoncological patients, for example, have resistance rates to methicillin of approximately 70% to 80%, with VRE being responsible for up to 41.1% of all Gram-positive bacteremia [17]. In hemodialysis patients, MRSA is the main infectious agent [46], being responsible for almost 30% of all deaths [15,49].

According to what has been reported in the literature, daptomycin is used for the treatment of serious infections caused by Gram-positive bacteria, especially for those strains that present resistance to the usual therapeutic options [8,17,49]. However, reports of daptomycin resistance towards strains of *S. aureus* and *Enterococcus* spp. have been associated with a mutation in the *vraS* and *pitA* genes that generates alterations in three glycerophosphoryl diester phosphodiesterase proteins (GdpD), (LiaF, GdpD, and Cls) and additionally, a greater voltage difference across the cytoplasmic membrane, reducing binding to its site of action [52,53].

It is important to note that daptomycin undergoes significant changes in its PK/PD in populations with special clinical conditions [47], being associated with a variability of serum levels despite receiving the standard dose [6,7,8,18]. In this type of population, it is suggested to increase the dose up to >8 mg/kg/day if they present serious infections, and the performance of TDM is of great support to be able to optimize management, guarantee therapeutic efficacy, and decrease risks of side effects and bacterial resistance [17,18,23]. However, despite the recommendations in favor of the use of TDM in patients with daptomycin, controversy is evidenced by positions where it is recommended that TDM is not necessary for dose adjustment given that the PK/PD changes of daptomycin are minimal. In addition, there were others who maintained an impartial position for not being able to recommend or contraindicate TDM for patients with daptomycin use [6,15].

Daptomycin and linezolid are among the last available options for the treatment of resistant Gram-positive infections [54,55]. In the treatment of *Staphylococcus aureus* bacteremia, vancomycin TDM has been part of the standard of care [56]; this is in consideration of its NTI. Daptomycin is not different, and the use of high doses of daptomycin is increasingly needed in scenarios such as endocarditis [57], and these dosages are frequently accompanied by marked elevations of CPK, especially in critically ill patients or patients with chronic kidney disease [58]. In clinical practice, it is common that CPK elevation leads to treatment withdrawal with daptomycin. TDM would make the event less frequent, but additionally, in cases where toxicity occurs, TDM would facilitate dosage adjustment and eventually allow continuity of a drug that may be the last therapeutic option in many clinical settings [59].

Different bioanalytical methods have been developed and published for the detection and quantification of daptomycin in plasma. Despite finding great variability in the techniques and the populations studied, all methods are suitable for pharmacokinetic studies and performance of daptomycin TDM [27,32]. However, it should be taken into account that the routine implementation of TDM requires a change in clinical practice, as well as a monetary investment for human, scientific, and technological talent; therefore, a prior evaluation of the technique should be made for each institution and its population [21].

As with all reviews, this study had limitations. First, the articles found were few and with low quality of evidence. Second, the populations were very different between the methods used for TDM, probably leading to results with a wide range. The few studies found and the heterogeneity in study design and population are major limitations of this review, as it is difficult to compile and compare results from a small collection samples with potential sampling bias.

## 4. Material and Methods

### 4.1. Search Strategy

In November 2020, we chose to conduct a scoping review of the literature to provide an overview of the information available on therapeutic drug monitoring in hospitalized patients with daptomycin use. A research protocol was designed, which was approved by the research subcommittee of the Faculty of Medicine of the University of La Sabana, which was developed under the guidelines of the Joanna Briggs Institute for Panoramic Reviews [60].

We performed a bibliographic search through the following databases: PubMed, EMBASE, Web of Science, and Scopus. We used key terms without restriction of date or language. Keywords searches included *drug monitoring, therapeutic drug monitoring,* and *daptomycin*. Supplementary Material Table S1 shows the search strategies that were used for each of the databases. Additionally, a literature search was performed in Open Gray System information on Gray Literature in Europe (http://www.opengrey.eu (accessed on 15 January 2021)), without results.

### 4.2. Evaluation and Selection of Studies

Figure 1 summarizes the selection process that was carried out. Once the final references were obtained, they were classified in EndNoteX9 software by eliminating duplicates. Through the Rayyan QCRI platform (https://rayyan.qcri.org (accessed on 15 January 2021)), COR and LGP independently applied the study inclusion criteria from the titles and abstracts to later obtain the full texts of the articles for review. Discrepancies of the included studies were resolved through discussion and consultation with the co-authors.

### 4.3. Inclusion and Exclusion Criteria

Articles were included if they described aspects related to daptomycin TDM in hospitalized patients regardless of its indication, those that described clinical and bioanalytical methods of monitoring, control of its side effects, bacterial resistance, and/or therapeutic success. Exclusion criteria were based on studies that spoke of patients with outpatient medical management and therapeutic monitoring of antibiotics other than daptomycin.

### 4.4. Graphing the Data

Once we reviewed the articles and considered them eligible, we summarized the main research results of each study and organized them in a graph using the following titles: monitoring method, population (n), objective, type of study, bioanalytical and clinical results, and conclusions (Table 1).

### 4.5. Collecting, Summarizing, and Reporting the Results

From Table 1, the researchers (COR and LGP) summarized the results corresponding to each of the research objectives, organized them thematically, and described and discussed them in detail. Specifically, the findings of the study characteristics, including type of clinical or bioanalytical method used for drug monitoring, year of publication, type of study, and PK/PD values, were noted, and we summarized the results of the thematic analysis in a tabular format (Table 2) corresponding to the research objectives as described here.

## 5. Conclusions

The need to create worldwide strategies for the appropriate use of antibiotics is clear, and one of these is the performance of therapeutic drug monitoring (TDM). This type of method brings great benefits, especially to those patients with special clinical conditions. In our study, it was possible to show that performing TDM helps us find certain benefits, such as (1) maintaining plasma levels within the therapeutic range; (2) adjusting the dose in a timely manner, based on PK/PD goals; and (3) controlling side effects and the development of bacterial resistance. However, given the variability in the different published methods, the difficulty of implementing several of these (the need for financial, scientific, and human resources), and the scarcity of evidence, more studies are required—probably controlled clinical trials (CCT)—to establish a clear therapeutic range, adequately documenting the clinical impact on the patients studied in order to develop a rapid, accurate, and clinically validated monitoring model that can be established for the routine treatment of those patients with serious infections, and which can also contribute to the evolution of personalized medicine.

Due to the aforementioned implications, such as an NTI in some indications, the clinical impact on infections with few treatment options, and little research, we consider that daptomycin is a molecule that should be investigated further in order to achieve TDM methods that will be commercially available.

## Figures and Tables

**Table 1:** Description of selected studies.

Monitoring Method	Population	Objective	Type of Study	Results	Conclusions	Reference
LC-MS/MS	Population n = 6, critically ill patients	Develop of a method for the quantification and analysis of daptomycin in a dry blood stain (DBS).	Bioanalytical methodology	RT: Daptomycin: 4.57 min -Average recovery of daptomycin extraction by DBS: 82.48–97.72% -V_1/2_: 11.56 ± 3.31 h -C_max_: 109.15 ± 56.39 mg/L -Vd: 87.65 ± 42.66 mL/kg -CL: 9.05 ± 5.79 mL/h/kg -AUC_24h_: 786.93 ± 451.65	Allows non-invasive sampling, micro-volume blood sample, useful for PK study in critically ill patients.	[16]
	Population n = 8, human plasma samples	Develop an LC-MS/MS method for the simultaneous quantification of 3 antibiotics.	Bioanalytical methodology	-Running time: 5.5 min -Accuracy: 95.9–116.6% -C_min_: 3.125 mg/L for daptomycin -Determined Conc.: 40 and 105 mg/L	TDM is useful in intensive care units and other pharmacokinetic studies.	[36]
	Population -Age: 54 years -Weight: 94 kg -Backg: hemodialysis (HD) without residual kidney function -Patho: sepsis after insertion of a pacemaker	Describe a case that required administration of daptomycin at the intraperitoneal level due to bacteremia by Gram-positive bacteria. Modify dose guided by TDM.	Case report	-TDM was performed: before, 4 h after, and 24 h after the 1st dose -C_max_: 4 h -At 24 h: daptomycin CL from intraperitoneal dialysis was determined -Daptomycin: 5.3 mg/kg every 48 h reached C_max_ -The dose was reduced to 4.3 mg/kg every 48 h and then to 3.2 mg/kg every 48 h -The target C_min_ and C_max_ were reached on day 22 -The dose was maintained at 300 mg (3.2 mg/kg) intraperitoneally every 48 h until day 32	The use of intraperitoneal daptomycin may be a possible therapeutic option when there is vascular deficit. TDM can help with serum concentration measurement and dose adjustments to maintain non-toxic concentration.	[29]
	N/A	Describe basic TDM concepts, clinical application examples, and the benefit of LC-MS/MS techniques.	Review article	-Measurement of drugs in blood should not replace monitoring with clinical or biological biomarkers -TDM is intended to allow dose adjustment to improve efficacy and prevent toxicity	TDM can contribute to the optimization and individualization of multiple drug dosing.	[24]
	Population n = 86 patients divided into 4 groups: 1. CrCl > 30 mL/min 2. CrCl ≤ 30 mL/min without RRT 3. End-stage kidney disease with intermittent hemodialysis 4. Renal failure with continuous RRT	Describe the variability in daptomycin exposure as a routine treatment in a clinical environment.	Bioanalytical methodology	-C_min_ was between 2 and 68 mg/L (median, 16.7 mg/L) -C_max_, between 20 and 236 mg/L (median, 66.2 mg/L) -Concentrations were frequently detected in patients with *E. faecium* and *Staphylococcus* coagulase (−). -Only 37 patients received daptomycin monotherapy -Average dose: 6 mg/kg -~40 patients received high doses: 6–13.8 mg/kg	Plasma concentration of daptomycin is often unpredictable, given by highly variable drug exposure that is only explained by the administered dose and renal function.	[40]
	Population n = 19, patients with peritoneal dialysis	Develop a method for the determination of daptomycin in peritoneal fluid, blood plasma, and urine.	Bioanalytical methodology	Concentration ranges: -Peritoneal fluid: 0.66 to 246 µg of mL -Plasma: 0.55 to 82.23 µg/mL -Urine: 5.2 to 23.68 g/mL	Economic, simple, rapid, and sensitive method. Method useful for monitoring and its application in pharmacokinetic studies.	[34]
	Population n = 1 patient, 3 plasma samples	Develop a bioanalytical method (LC-MS/MS) for the quantification of daptomycin plasma concentration in patients with severe infection.	Bioanalytical methodology	-C_max_ (30 min): 39 mg/L (>80 mg/L healthy patient) -C_min_ (24 h): 14 mg/L (9 mg/L healthy patient) -Dose increased due to persistent bacteremia to 12 mg/kg -C_max_ (30 min): 54 mg/L (>80 mg/L healthy patient) -No evidence of side effects	Good alternative to previously proposed HPLC-UV or LC-MS methods. Well suited to analysis time, range of measured concentration, precision, and accuracy.	[44]
	Population n = 30 patients	The modern LC-MS/MS has opened a new era for measuring antibiotic levels in blood or tissues in the hospital.	Poster	-It was possible to report the concentration of medications in less than 24 h -It has been used so far in 30 patients in whom the doctor considered vital the monitoring of the concentration of the drug	The use of bioanalytical methods to perform TDM still requires many studies. The impact on mortality cannot be assessed.	[25]
HPLC	Population n = 63, hospitalized patients receiving daptomycin	Assess the dose of daptomycin in a real-life study, variability between patients and clinical impact.	Observational prospective study	-Great inter-individual variability; C_min_: 10.6 mg/L (1.3–44.7 mg/L); C_max_: 44.0 mg/L (3.0–93.7 mg/L) -Adequate dose: 10 mg/kg for endocarditis	Daptomycin TDM optimizes management and prevents toxicity.	[18]
	Population n = 3 plasma samples Temperature T = −80 °C, −20 °C and 4 °C	Evaluate stability of daptomycin in blood at different temperatures.	Bioanalytical methodology	-Loss of concentration of daptomycin in plasma <10% after 6 months at T: −80 °C and −20 °C -Concentration of daptomycin at 4 °C, decreased more than 70% over 6 months -Concentration of daptomycin at body T (35 °C, 37 °C and 39 °C) decreased >50% after 24 h	Perform level measurement immediately. Daptomycin levels in plasma are stable at T < 20 °C.	[39]
	Population n = 35 patients hospitalized	Determine the pharmacokinetic profile of daptomycin.	Non-randomized clinical study	-No patient presented increased CPK -Vd: 0.5 L/kg -Renal function is the main cause of changes in daptomycin clearance V_1/2_: 9 h AUC/MIC: 692 ± 210 (6 mg/kg/day) 903 ± 280 (8 mg/kg/day)	TDM is needed secondary to a variation of PK/PD of daptomycin in hospitalized patients.	[19]
	Population n = 760 plasma samples from 168 patients from 50 hospitals	Provide a TDM service for daptomycin in the UK for 4 years.	Poster	-During 4 years (2007–2011) we received 760 serums samples for daptomycin TDM	~40% of the samples presented plasma concentration outside the range. -low concentration were more common in patients <18 years. -high concentration were similar in all ages. It is important to perform TDM in patients with daptomycin to optimize treatment.	[26]
	Population n = 9 patients Backg: requiring continuous veno-venous hemodiafiltration (CVVHDF)	Determine the pharmacokinetics of daptomycin at a dose of 6 mg/kg and the profiles of conc./t after multiple doses. Compare with current literature to develop robust recommendations regarding daptomycin dosage.	Bioanalytical methodology	-Daptomycin 8 mg kg every 48 h resulted in adequate levels without accumulation -Accumulation: when administered every 24 h -Clearance: 6.1 ± 4.9 mL/min -t_1/2_: 17.8 ± 9.7 h C_max_: day 1: 62.2 ± 16.2 mg/L day 2: 66.1 ± 17.3 mg/L day 3: 78.5 ± 22.1 mg/L C_min_: >4 mg/L (relevant bacteria were susceptible to daptomycin)	For critically ill patients, a daptomycin dose of 8 mg/kg is recommended, and TDM should be performed if possible.	[45]
	Population -Daptomycin: n = 5 patients -Linezolid: n = 14 patients Dose -Daptomycin: 6–8 mg/kg in 30 min -Linezolid: 600 mg every 12 h	Create and validate a sensitive, specific and reliable HPLC method to monitor plasma concentration of daptomycin in patients affected by severe infections caused by Gram-positive bacteria.	Bioanalytical methodology	Retention times: -Daptomycin: 17.4 ± 0.20 min -Accuracy values < 20% -C_max_: 52.8–84.7 mg/L -C_min_: 12.5 ± 3.1 mg/L (associated with reduced risk of increased CPK) -C_max_/MIC: 106–169	Robust and reliable method. Useful to measure concentration plasma daptomycin and linezolid.	[8]
	N/A	Develop an HPLC method to measure plasma concentration in patients with multiorgan failure.	Letter to the Editor	-Daptomycin retention time: ~5 min -Daptomycin: stable after three cycles; 99.1% for the 5 mg/L sample, 102.1% for the 20 mg/L sample and 98.4% for the 100 mg/L sample	Daptomycin stable after freeze/thaw cycles. Ideal method for laboratories requiring a fast response time of the test.	[22]
TDM	Population Age: 59 years Patho: bacteremia due to MRSA secondary to CVC	Present a case of a patient with bacteremia due to MRSA secondary to CVC, with suboptimal doses of daptomycin.	Case report	-C_max_: 12.2 mg/L (recommended level: 98.6 ± 12 mg/L) -C_min_: 2.5 mg/L (recommended level: 9.4 ± 2.5 mg/L) -A dose was given below the recommended: 3.28 mg/kg (recommended dose: 6 mg/kg)	TDM is essential for treatment optimization. TDM prevents the development of side effects, therapeutic failure, and the appearance of bacterial resistance.	[30]
	N/A	Describe the analytical methods used for the analysis of daptomycin.	Review article	-There are few studies demonstrating analytical methods for the measurement of daptomycin -Most of the studies described are HPLC and UHPLC	TDM is important for special populations More research is required to contribute to a better understanding of this problem and improve the therapeutic response.	[47]
	N/A	Review available evidence in relation to TDM of antibiotics. Describe how TDM can be used to positively improve outcomes for critically ill patients.	Review article	-Information on TDM with daptomycin is limited -High protein binding and variable renal clearance make it a candidate for TDM to achieve PK/PD target -It can help reduce the risk of rhabdomyolysis associated with levels of C_min_ > 24.3 mg/L, and when using doses higher than recommended	Critically ill patients with sepsis, skin burns, hypoalbuminemia, and requiring RRT would benefit from TDM. TDM works as a mechanism to reduce side effects and optimize doses.	[48]
	Population Age: 63 years Backg: morbid obesity and CKD Patho: cellulitis	Describe a case of severe cellulitis, treated with high doses of daptomycin. The dose of daptomycin was optimized by TDM.	Case report	Dose adjustment by TDM: -Daptomycin: 1200 mg/48 h for 30 min→1200 mg/36 h for 30 min -Meropenem: 0.25 mg every 8 h for 6 h→500 mg every 4 h CI -Favorable clinical response at 72 h -Increased CPK for which it was thought to suspend daptomycin	TDM in infected patients can help optimize doses for therapeutic success.	[28]
	N/A	Define role of TDM in antibiotic dosing.	Review article	-There are no controlled clinical trials that demonstrate a reduction in mortality from the use of TDM -There are publications that suggest that the use of TDM is probably beneficial to patients	TDM serves as a method to adjust the dose of some particular antibiotics in the relevant population.	[23]
UHPLC-MS/MS	Population n = 2 patients	Develop and validate quantitative method to measure total and free daptomycin in human plasma using UHPLC-MS/MS.	Bioanalytical methodology	-Retention time: 2.17 min -The concentration range of the calibration curve is 0.5 to 200 g/mL, wider than in all previous reports -C_max_ and C_min_ were reached -Binding to plasma proteins: 97.5–97.9%	Appropriate method for quantifying total concentration plasma and concentration of free daptomycin. Calibration curves with wide ranges capable of measuring C_max_ and C_min_. Short measurement and analysis time, accuracy, selectivity, stability, and recovery rate.	[41]
	Population n = 6, plasma from humans	Provide a simple, fast, and accurate quantification of the concentration of 4 antibiotics.	Bioanalytical methodology	-AUC/MIC daptomycin: 75 and 537 for efficiency -C_max_/MIC: 12 and 94 according to bacterial species -Main marker of skeletal muscle toxicity: CPK elevation (relation with C_min_ > 24.3 mg/L)	Method is a good option for prospective PK studies and simultaneous monitoring of anti-MRSA drugs.	[35]
	Population n = 6 human plasma samples	Develop a rapid LC-MS/MS method for the simultaneous quantification of 5 antibiotics.	Bioanalytical methodology	-Extraction recovery time: 79.3% to 105.9% -Intra- and inter-assay coefficients of variation: 1.95–12.77%, 2.56–8.16%, and 2.12–11.38% for low, medium, and high levels	Fast and robust method. Useful for routine TDM and optimization of resource use.	[37]
	Population n = 6 blood samples	Develop a bioanalytical method to determine concentration of daptomycin at the plasma level.	Bioanalytical methodology	-Concentration of daptomycin in patient No. 3: 6.8 µg/mL -Accuracy in concentration low, medium, and high of the eight target peaks was: 89.3–110.7% -LOD were <70 ng/mL	Sensitive and selective method. Analysis time: 10 min. Method indicated for the realization of TDM.	[42]
HPLC-UV	Population n = 10 plasma samples	To develop and validate a new chromatography method for the measurement of plasma concentration of daptomycin. Compare method with commercially available LC-MS/MS.	Bioanalytical methodology	-Daptomycin: C_max_: 5.8 min after injection -Inter and intraday coefficients of variability < 15% -Comparison with a commercially available reference LC-MS/MS method showed an excellent correlation (r^2^ = 0.9474)	-Precise and reproducible method. Quantifies plasma concentration of daptomycin within the min–max range of values expected after drug administration at prescribed dose.	[38]
	Population n = 30, hemato-oncological adult patients	Evaluate if adequate exposure and pharmacodynamic objectives are achieved in a cohort of hemato-oncological patients with conventional dose.	Observational prospective study	-Dose 6 mg/kg/day: optimal PTA (probability of reaching objective) ≥ 80%, if pathogens with MIC up to 0.25 mg/L -Higher doses: up to 12 mg/kg/day needed to achieve goal if pathogens with MIC 0.5 mg/L in all other scenarios -CL: 0.56 L/h in bone and joint infections; 1.81 L/h in sepsis and bacteremia due to *S. aureus*	Considering daptomycin doses ≥ 8 mg/kg/day in various onco-hematological patients.	[17]
HPLC-UV and LC-MS/MS	Population Studies in critically ill adult patients	Review available antibacterial, antifungal and antiviral TDM data. Recommend how to use.	Review article	-AUC/MIC >666 for efficiency -C_min_: ≥24.3 mg/L is associated with a greater probability of elevated CPK	Performing TDM is the only safe and effective way to ensure that critically ill patients achieve adequate therapeutic levels. Controlled studies focused on clinical outcomes are needed to justify routine use of TDM.	[6]
	Population -Critically ill patients	Review the available evidence for anti-infective TDM. Describe its use to optimize the treatment of critically ill patients.	Review article	-Bactericidal activity of daptomycin: AUC_0–24_/MIC: 38–442, > AUC_0–24_/MIC efficacy > 666 -In vitro: AUC_0–24_/MIC ≥ 200 resistance suppression -C_max_/MIC: 12–94 optimal bacteriostatic effect -C_min_ > 24.3 mg/L (probability of CPK elevation)	TDM could be useful to optimize treatment in critically ill patients.	[7]
UHPLC-PDA	Population n = 46 patients, 52 cases (6 patients received multiple courses of treatment)	Assess the clinical importance of Cmin in relation to the safety of daptomycin use.	Observational retrospective study	-C_min_ > 24.3 mg/mL in 7 cases (none had elevated CPK) -CPK elevation: 2 patients (none needed treatment interruption) -Treatment interruption: 4 patients, suspected adverse effects; C_min_: 8.6 and 8.1 mg/L -2 patients: doses 9.4 and 10.0 mg/kg; C_min_ equal to dose < 9 mg/kg	Daptomycin is safe The level of C_min_ of 24.3 mg/L is not considered clinically significant for the elevation of CPK, adverse effects, or treatment interruption. C_min_ levels > 24.3 mg/L are suggested. Measurement of serum levels is useful for cases of subtherapeutic levels in standard dose treatment.	[49]
	Population n = 44 patients	Development and validation of a method to measure daptomycin in plasma and dry plasma spots (DPS).	Bioanalytical methodology	-Stability: DPS 7 days at room temperature, 30 days at 4 °C -Retention times: 4.70 (±0.05) for daptomycin -Average recovery of plasma: 94.48%	DPS—Safe and economical option for storing and shipping plasma samples, suitable for daptomycin PK and TDM studies in hospitals without a monitoring laboratory. UPLC versus HPLC, allows a shorter analysis time, greater reproducibility, and sensitivity.	[32]
HPLC-MS	Population Patients being treated for serious bacterial infections	Develop an HPLC-MS/MS method to quantify plasma concentration of 12 antibiotics.	Bioanalytical methodology	-TDM-guided dose titration in renally impaired daptomycin patients may prevent toxic rhabdomyolysis	Method developed makes it possible to adequately quantify the plasma concentration of 12 antibiotics.	[33]
	Population n = 7, blood plasma samples	Development and validation of a method for the simultaneous extraction and quantification of daptomycin.	Bioanalytical methodology	-Retention time: 10.00 ± 0.25 min for daptomycin -No interference by other medications -Average recovery: 90.5% -Stability: room temperature for 8 h	TDM for antibacterial could be useful in special populations. Rapid, specific, sensitive, accurate, and reproducible method to measure daptomycin in human plasma.	[31]
LC-MS/MS with core–shell octadecylsilyl microparticle.	Population n = 5 plasma samples	Develop an LC-MS/MS method to quantify total free daptomycin in plasma in infected patients.	Bioanalytical methodology	Lower limits: -Total daptomycin: 1.0 mcg/mL -Free daptomycin: 0.1 mcg/mL Plasma concentration ranges: -Total daptomycin: 3.01–34.1 mcg/mL -Free daptomycin: 0.39–3.64 mcg/mL -Binding to plasma proteins: 80.8–94.9%	Acceptable method for monitoring the pharmacokinetics of daptomycin in infected patients.	[20]
Bayesian estimation	N/A	Describe methods for antibiotic dose optimization, focus on Bayesian programs.	Review article	-Faster target concentration reach and in >% of patients with Bayesian method -Optimizing exposure to antibiotics gives better clinical results	Bayesian estimation methods are support programs. With PK/PD and clinical experience they are helpful for dose optimization.	[21]
MCS–TDM	Population n = 16 patients with MRSA infections	Explore the optimal daptomycin dose regimen Determine the need and validation of a high dose regimen from PK/PD parameters using Monte Carlo simulation and TDM.	Bioanalytical methodology	-Volume of distribution: 0.13 ± 0.012 L/kg (greater than that of healthy volunteers) -t_1/2_: 8.9 and 34.9 h (prolonged as creatinine clearance decreased) -MCS: the cumulative fraction of dose response: 6 mg/kg every 24 h: -C_max_/MIC > or equal to 60: 72% AUC/MIC > or equal to 666: 78.8% 10 mg/kg every 24 h: both 99% TDM: dose of 6 mg/kg every 24 h -Patients who reached the peak, and the AUC was: 40% (2 of 5 patients)	Intra-individual variability may indicate the need for TDM. A high dose regimen (>8 mg/kg) may be necessary to ensure treatment efficacy.	[43]
Population PK model	Population n = 26, patients with hemodialysis 3 times a week	Recommend doses in a standard HD schedule of three times a week for each interdialytic period.	Review article	-Interdialytic period 72 h: increase of 50% (dose >: C_min_ 24.3 mg/L) -Interdialytic period 48 h: same dose (4–6 mg/kg) intra or post-HD -No patient presented elevations of CPK	More studies are needed. Intensive CPK monitoring is justifiable.	[46]
Dosing Protocol	Population n = 183 patients with positive cultures for VRE	Evaluate changes in daptomycin dose and adherence to the daptomycin dosing protocol and safety guidelines.	Observational retrospective study	-Average dose increased from 453 ± 144 mg to 571 ± 208 mg -Dose/weight increased 6.1 ± 1.4 mg/kg to 7.6 ± 1.6 mg/kg -Post-protocol: dose >8 mg/kg went from 4% to 52% -Dose <8 mg/kg, increased by 30% to >8 mg/kg -CPK increase: 43% to 64% -Weekly CPK monitoring for doses > 8 mg/kg	A dosing protocol requires closer monitoring to avoid the development of side effects.	[50]
N/A	Population -Age: 45 years -Backg: obesity -Patho: bacteremia	Prove the importance of TDM in the antimicrobial therapy of special populations.	Case report	-Blood cultures persisted positive after 14 days -Management was adjusted and TDM of daptomycin was started -The effective doses were 14 mg/kg of lean body weight for daptomycin	Multidisciplinary management based on TDM in the antimicrobial treatment of special populations was demonstrated.	[27]
N/A	Population -n = 122 -Spanish patients admitted to ICU	Describe the characteristics of daptomycin in Spanish ICU patients.	Review article	-Use: 85.7% of cases as rescue treatment -Dose: 6 mg/kg/day in 52% -Duration: 10.2 days -Overall clinical efficacy of 73.7%	It is a new option and a good alternative for the treatment of serious Gram-positive infections in critically ill patients.	[15]

LC-MS/MS: liquid chromatography–tandem mass spectrometry; HPLC: high performance liquid chromatography; TDM: therapeutic drug monitoring; UHPLC-MS/MS: ultrahigh pressure liquid chromatography–tandem mass spectrometry method; HPLC-UV: high performance liquid chromatography with ultraviolet detection; UHPLC-PDA: ultrahigh performance liquid chromatography equipped with a photodiode array; HPLC-MS: high performance liquid chromatography–tandem mass spectrometry; MCS-TDM: Monte Carlo simulation and therapeutic drug monitoring; CPK: creatine phosphokinase; RRT: renal replacement therapy; CL: clearance; CKD: chronic kidney disease; MRSA: methicillin-resistant *Staphylococcus aureus*; CVC: central venous catheter; LOD: limit of detection; Conc: concentration, t_1/2_: half-life.

**Table 2:** Tabular format of results.

Parameter	Results
No. of publications per year	2008: 1 [22] 2009: 1 [25] 2010: 5 [8,15,35,37,46] 2011: 2 [30,44] 2012: 3 [30,47,51] 2013: 6 [23,32,36,39,44,48] 2014: 1 [48] 2015: 3 [38,42,49] 2016: 3 [7,25,33] 2017: 2 [17,39] 2018: 4 [16,18,22,24] 2019: 4 [26,43,45,50] 2020: 4 [6,21,40,52]
Study design type	Letter to the editor: 1 [22] Poster: 2 [25,26] Case report: 4 [27,28,29,30] Bioanalytical method: 18 [8,16,20,31,32,33,34,35,36,37,38,39,40,41,42,43,44,45] Review article: 9 [6,7,15,21,23,24,46,47,48] Observational prospective study: 2 [17,18] Observational retrospective study: 2 [49,50] Non-randomized clinical study: 1 [19]
Therapeutic drug monitoring method	1. Liquid chromatography–tandem mass spectrometry (LC-MS/MS): 8 articles [16,22,25,29,33,37,42,46] 2. High-performance liquid chromatography (HPLC): 7 articles [8,18,23,28,30,41,47]. 3. Therapeutic drug monitoring (TDM): 5 articles [19,27,32,34,49]. 4. Ultrahigh pressure liquid chromatography–tandem mass spectrometry method (UHPLC-MS/MS): 4 articles [38,39,43,44]. 5. Ultrahigh-performance liquid chromatography with UV detection (HPLC-UV): 2 articles [17,38]. 6. High performance liquid chromatography with ultraviolet detection (HPLC-UV) and liquid chromatography–tandem mass spectrometry (LC-MS/MS): 2 articles [6,7]. 7. Ultrahigh-performance liquid chromatography equipped with a photodiode array (UHPLC-PDA): 2 articles [32,49]. 8. Liquid chromatography using a core-layer octadecylsilyl microparticle coupled to tandem mass spectrometry: 1 article [20]. 9. Bayesian estimation 1 article [21]. 10. Dosing protocols: 1 article [50]. 11. Monte Carlo and TDM simulations: 1 article [43]. 12. Population PK models: 1 article [46].
C_max_	58.9 mg/L [49] 109.15 mg/L [16] 52.8 mg/L [8] 66.2 mg/L [40] 62.2 mg/L [6] 66.1 mg/L [6] 78.5 mg/L [6] Average: 70.55 mg/L
C_min_	18.6 mg/L [49] 12.5 mg/L [8] 16,7 mg/L [40] 14 mg/L [44] 4 mg/L [45] Average: 13.16 mg/L
AUC/MIC	786.93 ± 451.65 [19] Dose: 6 mg/kg/day [19]: 692 ± 210 406.09 ± 175.2 510.1 ± 129.3 962.6 ± 225.3 Average: 642.69 Dose: 8 mg/kg/day [19] 903 ± 280 (8 mg/kg) [18] 654.5 ± 312.7 584.3 ± 367.2 1013.6 ± 324.9 Average: 788.85

## Data Availability

Data is contained within the article or supplementary material.

## Supplementary Materials

The following are available online at https://www.mdpi.com/2079-6382/10/3/263/s1, Table S1: Search strategy.
