# Supplementary material for: Impact on Antibiotic Resistance, Therapeutic Success, and Control of Side Effects in Therapeutic Drug Monitoring (TDM) of Daptomycin: A Scoping Review
Source: Antibiotics (Basel). 2021 Mar 5;10(3):263. doi: 10.3390/antibiotics10030263 (PMC8001274; doi:10.3390/antibiotics10030263)
Supplement: Supplementary file 1 [file antibiotics-10-00263-s001.pdf]

Impact on Antibiotic Resistance, Therapeutic Success, and Control of Side Effects in Therapeutic Drug Monitoring (TDM) of Daptomycin: A Scoping Review

Carolina Osorio [1], Laura Garzón [1], Diego Jaimes [1], Edwin Silva [2] and Rosa-Helena Bustos [1,*]

[1] Evidence-Based Therapeutics Group, Clinical Pharmacology, Universidad de La Sabana, Chía 140013, Colombia; carolinaosre@unisabana.edu.co (C.O.), lauragapa@unisabana.edu.co (L.G.); diegojf@unisabana.edu.co (D.J.)
[2] Faculty of Medicine, University of La Sabana, Chía 140013, Colombia; edwin.silva@unisabana.edu.co
* Correspondence: rosa.bustos@unisabana.edu.co; Tel.: +57-1-8615555

Supplementary Materials

Table S1. Search strategy

Database	Indication	Search Strategy	Results
**Pubmed**	Therapeutic Drug Monitoring during Daptomycin use	("Drug Monitoring"[Mesh]) AND "Daptomycin"[Mesh]	18
		therapeutic drug monitoring AND daptomycin	129
		therapeutic drug monitoring [title] AND daptomycin [title]	3
		daptomycin AND therapeutic drug monitoring	129
		Daptomycin [title] AND therapeutic drug monitoring [title]	3
		drug monitoring AND daptomycin	129
		(drug monitoring OR therapeutic drug monitoring OR TDM) AND daptomycin	130
**EMBASE**	Therapeutic Drug Monitoring during Daptomycin use	Quick Search: ('therapeutic drug monitoring'/exp OR 'therapeutic drug monitoring' OR (therapeutic AND ('drug'/exp OR drug) AND ('monitoring'/exp OR monitoring)) OR 'drug monitoring'/exp OR 'drug monitoring' OR (('drug'/exp OR drug) AND ('monitoring'/exp OR monitoring)) OR tdm) AND ('daptomycin'/exp OR daptomycin)	449
		All fields: (therapeutic AND drug AND monitoring OR (drug AND monitoring) OR tdm) AND daptomycin	447
		('therapeutic drug monitoring':ti OR 'drug monitoring':ti OR tdm:ti) AND daptomycin:ti	8
		('therapeutic drug monitoring':ti,ab,kw OR 'drug monitoring':ti,ab,kw OR tdm:ti,ab,kw) AND daptomycin:ti,ab,kw	66
		('therapeutic drug monitoring':ab,ti OR 'drug monitoring':ab,ti OR tdm:ab,ti) AND daptomycin:ab,ti	54
		TOPIC: (therapeutic drug monitoring) AND TOPIC: (daptomycin)	154

**Web of Science**	Therapeutic Drug Monitoring during Daptomycin use	TITLE: (therapeutic drug monitoring) AND TITLE: (daptomycin)	5
		TITLE: (drug monitoring) AND TITLE: (daptomycin)	5
		TOPIC: (drug monitoring) AND TOPIC: (daptomycin)	111
		TITLE: (TDM) AND TITLE: (daptomycin)	0
		TOPIC: (TDM) AND TOPIC: (daptomycin)	11
		TOPIC: ((therapeutic drug monitoring OR drug monitoring OR TDM)) AND TOPIC: (daptomycin)	112
**Scopus**	Therapeutic Drug Monitoring during Daptomycin use	(TITLE-ABS-KEY (therapeutic AND drug AND monitoring) AND TITLE-ABS-KEY (daptomycin))	129
		(TITLE-ABS-KEY (drug AND monitoring) AND TITLE-ABS-KEY (daptomycin))	415
		(TITLE (drug AND monitoring) AND TITLE (daptomycin))	4
		(TITLE (therapeutic AND drug AND monitoring) AND TITLE (daptomycin))	4
		(TITLE (therapeutic AND drug AND monitoring) AND TITLE-ABS-KEY (daptomycin))	17
		(TITLE-ABS-KEY (therapeutic AND drug AND monitoring) AND TITLE (daptomycin))	45
		(TITLE-ABS-KEY (tdm) AND TITLE-ABS-KEY (daptomycin))	22
		(TITLE (tdm) AND TITLE (daptomycin))	1
**OpenGrey**	Therapeutic Drug Monitoring during Daptomycin use	therapeutic drug monitoring AND daptomycin	0
		**Total**	**2600**
